# Optimizing Surgery for Idiopathic Scoliosis: Does a Dual Approach Help Young Surgeons?

**DOI:** 10.7759/cureus.75745

**Published:** 2024-12-15

**Authors:** Bryan O Ren, Sunita R Mengers, Ryan J Furdock, R Justin Mistovich, Jonathan E Belding

**Affiliations:** 1 Department of Orthopedic Surgery, University of Michigan, Ann Arbor, USA; 2 Department of Orthopedic Surgery, University Hospitals Cleveland Medical Center, Cleveland, USA; 3 Department of Orthopedic Surgery, MetroHealth Medical Center, Cleveland, USA

**Keywords:** adult idiopathic scoliosis, dual attending, scoliosis, two attending, young surgeons

## Abstract

Introduction

In idiopathic scoliosis surgery, studies have shown two attending surgeons have better curve correction, pain, and recovery time. There is conflicting evidence on operative time, blood loss, infection rate, and hospital length of stay. Limited literature examines the impact of surgeon experience on the dual approach. The purpose of this study was to evaluate the performance of two young orthopedic attendings compared to a senior-level attending in idiopathic scoliosis surgery.

Methods

We examined adolescent idiopathic scoliosis (AIS) patients undergoing posterior spinal fusion by a single or dual-attending approach. We performed a 1:1 propensity score match with the variables of age at surgery, sex, body mass index, Cobb angle, and number of levels fused. Peri- and postoperative outcomes were analyzed.

Results

There were 24 patients in each cohort. Patients having surgery by dual attendings had a shorter mean operative time overall (232 vs. 327 minutes, p<0.001) and per levels fused (19 vs. 26 minutes per level, p<0.001). Dual attendings had better percent curve correction (70% vs. 56%, p=0.001) and smaller overall final postoperative curve magnitude (17° vs. 25°, p<0.001). Estimated blood loss (421 vs. 989 mL, p=0.023) and cell saver volume transfused (59 vs. 178 mL, p<0.001) were lower in dual attending cases. Dual attending patients had a shorter length of stay (3 vs. 4 days, p<0.001). There were no differences in Hemovac blood loss, transfusion requirements, need for intensive care, or complications. Over time, the young dual attendings improved in hospital length of stay (R: -0.617, p=0.001) and hemovac blood loss (R: -0.474, p=0.019).

Conclusion

A dual attending approach in idiopathic scoliosis surgery may result in shorter operative time, greater curve correction, reduced operative blood loss, and shortened hospital length of stay. No differences were identified in postoperative blood loss or transfusion requirement, need for intensive care, or overall complication rate. Within the limitations of this study, we conclude that dual attending surgery in idiopathic scoliosis is safe and effective when conducted by two young orthopedic surgeons, with results that are similar to that of a more experienced senior surgeon.

## Introduction

Adolescent idiopathic scoliosis (AIS) is a common condition with an estimated prevalence of 5.2-8% [1]. While most cases of idiopathic scoliosis (IS) are mild and can be managed nonoperatively, operative treatment is indicated for larger magnitude curves to prevent future complications such as progression of the curve, cardiopulmonary dysfunction, and patient psychological distress due to deformity [2-4]. Posterior spinal fusion (PSF) is the preferred method of operative correction for IS. However, PSF for pediatric spinal deformity is a complex procedure, with typical operative times of four hours or greater, as well as risks of considerable intraoperative blood loss, infection, neurologic injury, and implant failure [5-7]. When present, these complications often result in substantial patient morbidity and are accompanied by an extended hospital length of stay and increased financial burden [8].

Prior investigations have examined the utility of dual-attending surgery in the operative correction of IS when compared to a single-attending approach [9-11]. Specifically, studies have demonstrated positive outcomes with a dual attending approach with regards to improved correction of preoperative curve, decreased narcotic consumption, and faster recovery time, though conflicting evidence remains in association with total operative time, intraoperative blood loss, or need for transfusion, infection rate, and hospital length of stay [9-11]. Additionally, limited studies have demonstrated a lengthy learning curve necessary to achieve optimal patient outcomes with dual attending operations for pediatric spinal deformity surgery [12].

Despite these potential benefits, there is a dearth of literature examining the role of surgeon experience as a variable impacting the potential value of a dual attending approach [12,13]. At our institution, two young orthopedic attending surgeons consistently work as a team in spinal deformity cases, while a previously established senior attending independently practices. While we suspected that the dual approach may have led to a more accelerated development of outcomes commensurate with a high volume, well-experienced senior surgeon, this was previously not formally studied either in the literature or at our institution. Therefore, we sought to analyze perioperative and postoperative outcomes of the dual attending operations for the correction of IS in comparison to those of a senior attending operating independently. In addition, we evaluated the dual attending approach in relationship to experience gained to assess for improvement in surgical outcomes. We hypothesize that dual attending surgery will result in improved outcomes with regard to operative time, blood loss, curve correction, and hospital length of stay. Additionally, we predicted improvement in similar parameters with increased experience with a dual-attending approach.

## Materials and methods

Following institutional review board approval (STUDY20211128 and IRB21-00483), a therapeutic retrospective cohort investigation was conducted of patients with IS undergoing PSF by either a single or dual attending approach. Inclusion criteria were all patients undergoing posterior spinal fusion for idiopathic scoliosis. Growing spine procedures, revision surgeries, and non-IS etiology patients were excluded. The dual attendings were both within their first decade of independent practice, and their cohort consisted of patients undergoing PSF for IS, with both surgeons functioning as co-attendings and present for the entirety of the case. The single attending had been practicing for several decades with a busy, established, academic spinal deformity practice. His cohort of patients included all first-time PSFs performed for IS within the final decade of practice. Both cohorts utilized Aquamantys and either tranexamic acid or aminocaproic acid in all cases. A standardized approach was utilized for dissection. The senior, single attending used two electrocautery devices and two suctions, with the surgeon and either senior resident or fellow simultaneously exposing. In contrast, the dual attendings performed a team dissection, with one utilizing two Cobb elevators to retract while the other utilized a Bovie and suction to expose, and then they switched roles after one side was exposed. 

Following a retrospective review, 24 patients were identified for the dual attending cohort with an index surgery from 2017-2021. A total of 175 patients were identified from the single attending cohort, with index surgery ranging from 2008-2017. A 1:1 propensity score match was performed with the single attending cases, so there were 24 patients each in the dual attending and single attending cohorts, respectively. The variables included in the propensity match were age at surgery, sex, body mass index, Cobb angle, and number of levels fused. Patient demographics, and perioperative and postoperative outcomes between the groups were analyzed via Chi-squared, Fisher's exact, and independent samples T-tests. Pearson's correlation coefficient was performed to determine trends within the dual attending cases. All statistical analyses were performed with the Statistical Package for the Social Sciences software for Mac version 28.0 (IBM Inc., Armonk, New York), with an alpha value of <0.05 to determine statistical significance.

## Results

The final dual-attending and single-attending cohorts each consisted of 24 patients following propensity score matching. The mean age of the dual and single attending cohort was 13.1±1.7 and 13.4 ±1.8 years old, respectively. Patients undergoing surgery by a dual attending approach had an overall shorter mean operative time (232 vs. 327 minutes, p<0.001) as well as shorter mean operative time per level fused (19 vs. 26 minutes per level, p<0.001; Table 1).

**Table 1:** Perioperative and postoperative characteristics between the dual young attending and one senior attending cohorts A p-value of <0.05 indicates statistical significance. Bold values indicate statistical significance. pRBC - packed red blood cells; FFP - fresh frozen plasma; PICU - pediatric intensive care unit; HCT - hematocrit test

Variable	Dual Young Attendings	One Senior Attending	p-value
Length of stay (days)	3.3 (±0.6)	4.23 (±0.6)	<0.001
Use of hooks	-	-	0.003
Yes	22	13	-
No	2	11	-
Number of screws	17.0 (±2.5)	11.8 (±7.6)	0.004
Estimated blood loss (mL)	420.8 (±295.0)	988.8 (±1150.0)	0.023
Hemovac blood loss (mL)	503.5 (±417.2)	586.5 (±248.5)	0.407
Cell saver (mL)	58.8 (±82.6)	177.9 (±115.2)	<0.001
pRBC transfused (units)	0.4 (±0.9)	0.7 (+1.5)	0.413
FFP transfused (units)	0.2 (±1.0)	0.2 (±0.8)	0.877
Platelets transfused (units)	0	0.1 (±0.4)	0.328
Albumin	-	-	0.009
Yes	17	8	-
No	7	16	-
Perioperative complications	-	-	0.489
Yes	0	2	-
No	24	22	-
Postoperative complications	-	-	1
Yes	1	2	-
No	23	22	-
PICU	-	-	0.489
Yes	0	2	-
No	24	22	-
Preop HCT	39.8 (±3.3)	41.2 (±2.8)	0.118
Preop Hgb	13.3 (±1.3)	13.8 (±1.2)	0.202
HCT POD1	30.2 (±5.3)	22.2 (±11.1)	0.004
Hgb POD1	10.2 (±1.9)	7.3 (±3.5)	0.001
HCT POD2	28.2 (±4.5)	25.4 (±8.3)	0.213
Hgb POD2	9.5 (±1.5)	7.3 (±3.5)	0.01
HCT POD3	29.1 (±4.8)	24.2 (±9.7)	0.113
Hgb POD3	9.8 (±1.6)	7.3 (±3.3)	0.023
Vancomycin powder	-	-	<0.001
Yes	24	5	-
No	0	19	-
Operative time (min)	232.5 (±58.5)	327.2 (±92.4)	<0.001
Operative time per level fused (min)	19.2 (±4.0)	26.1 (±6.9)	<0.001
Final postop curve (°)	16.7 (±7.2)	25.5 (±9.1)	<0.001
Percent curve correction (%)	70.5 (±11.8)	56.0 (±17.4)	0.001
Final months postop (months)	18.1 (±15.4)	49.4 (±28.8)	<0.001

There were no differences in mean upper or lower instrumented vertebra with the mean UIV and LIV being T3 and L2, respectively, in both cohorts. There also was no difference in postoperative Hemovac blood loss, blood product transfusion requirements, need for the pediatric intensive care unit, or perioperative or postoperative complications after one year.

**Figure 1:**
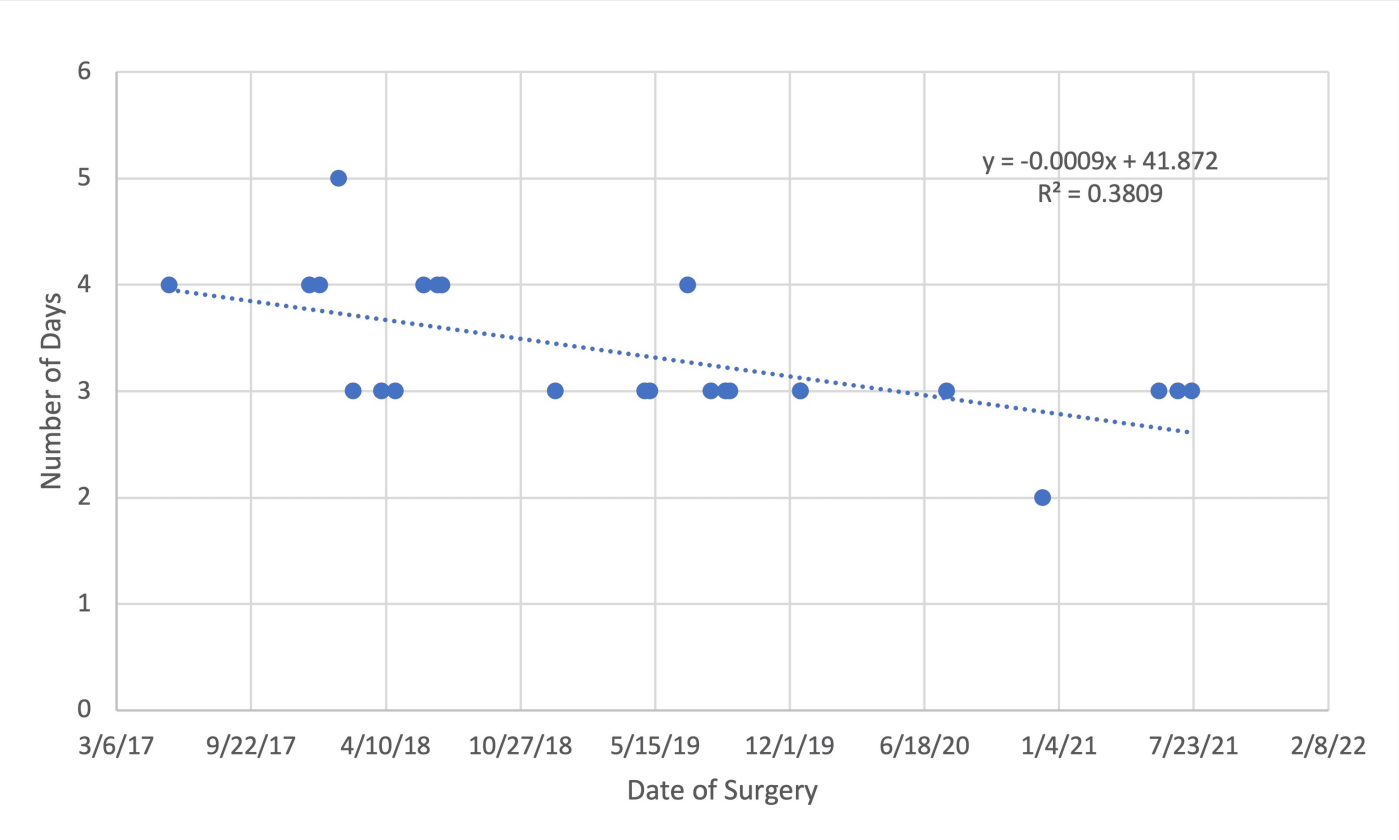
Trends in hospital length of stay over time in dual attending cases

**Figure 2:**
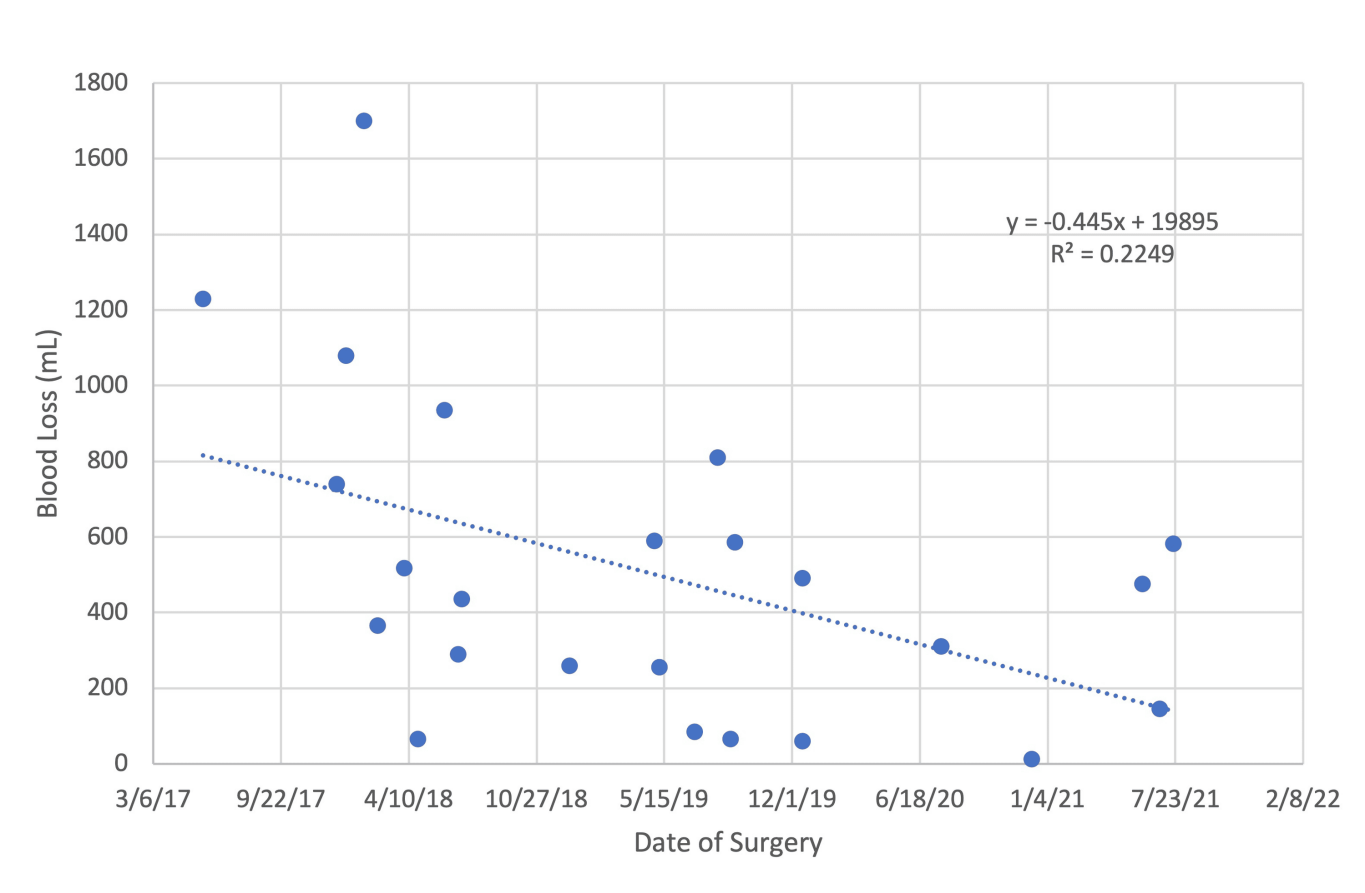
Trends in hemovac blood loss over time in dual attending cases

When evaluating outcomes of dual attending operations as the surgeons gained experience over time, there was an improvement in hospital length of stay (R: -0.617, p=0.001; Figure 1) and Hemovac blood loss (R: -0.474, p=0.019; Figure 2).

There were no differences in patient age at the time of operation, BMI, preoperative Cobb angle, number of levels fused, number of screws placed, estimated blood loss, amount of cell saver transfused, length of procedure, or final postoperative curve magnitude over time. 

## Discussion

The present study determined that a dual attending approach in IS surgery with young surgeons resulted in reduced operative blood loss, greater curve correction, shorter operative time, and shortened hospital length of stay compared to a single, senior surgeon. A number of surgeons did not impact postoperative blood loss or transfusion requirement, need for intensive care, or complication rates.

We found a significant reduction in intraoperative blood loss and quantity of blood salvaged with a dual attending approach with no differences in postoperative blood loss as measured by Hemovac drain output or need for transfusion. Known intraoperative risk factors for increased blood loss include prolonged duration of surgery, greater length of fusion, and elevated mean arterial pressure [14,15]. Additionally, an association exists between the need for allogenic blood transfusion and prolonged hospital length of stay, as well as increased risk of delayed postoperative infection following PSF for IS [16,17]. However, despite known risk factors and implementation of mitigating techniques, perioperative blood loss during PSF for IS remains a concern, with mean estimated blood loss of up to 1500 mL reported in some studies [9,18].

In an analogous study comparing a single versus dual attending approach for Lenke class 1 and 2 patients undergoing PSF for IS, Kwan et al. similarly reported significant improvements in intraoperative blood loss and reduced cell salvage for the dual attending cohort [9]. Likewise, Chan et al. reported greater blood loss for a single attending when prospectively compared to dual attendings performing PSFs for IS [19]. Furthermore, in a similar retrospective study, Halanski et al. reported no significant difference in intraoperative blood loss, but patients in the single attending cohort ultimately had a significantly greater postoperative decrease in hemoglobin from preoperative measurements [10]. All four investigations additionally reported significantly shorter operative times with a dual-attending cohort. However, the three prior studies found significantly increased requirements for allogenic blood transfusion with a single-attending approach compared to a dual-attending approach. This difference may be due to variations in hospital or attending policy related to transfusion threshold. Nevertheless, the general findings of reduced blood loss may be attributed, in part, to improved operative times as well as greater vigilance to achieve more rapid cautery with two attendings when compared to a single attending operating independently. Based on the findings of this investigation and prior studies, a dual-attending approach appears beneficial in reducing perioperative blood loss.

We also found improved percent curve correction from preoperative measurements, the overall magnitude of the postoperative curve, and a greater number of pedicle screws placed per operation. The dual attendings favored greater screw density in order to aim for increased curve correction. Our data is consistent with the findings of Halanski et al., who reported a significantly greater improvement in preoperative Cobb angle and a greater number of levels fused with a dual-attending approach [10]. A larger percentage of correction of preoperative Cobb angle has been previously correlated with greater improvements in Scoliosis Research Society-30 scores as well as greater patient satisfaction [20]. Therefore, improved curve correction secondary to a dual-attending approach may translate to clinically relevant improvements in patient outcomes, though further investigation is necessary to directly compare patient satisfaction with a dual versus single-attending approach.

As with any change in surgical care protocol, the financial costs must be weighed against the benefits of the modification. Multiple prior investigations have identified significantly shorter hospital length of stay amongst patients undergoing PSF for IS with a dual-attending approach [9-12]. The findings of our investigation are consistent with that of prior literature. Previously identified risk factors for increased length of hospitalization following PSF for IS include female sex, increased surgical time, greater fusion length, elevated intraoperative blood loss, need for postoperative blood transfusion requirement, higher narcotic demand, and development of wound complications [8,21,22]. In our investigation, reduced blood loss and surgical times likely contributed to shorter hospital stays, while postoperative need for transfusion was an additional factor in other studies. With an estimated additional cost of $5198 to $8825 per day of hospitalization, a reduction in hospital length of stay with a dual-attending approach may translate to a substantial reduction in healthcare expenditure [23,24]. However, additional factors must be considered. In a cost analysis of single attending versus dual attending involvement for radical cystectomy and urinary diversion procedures, the dual attending operations had significantly shorter operative time, which resulted in lower total operative and anesthesia material charges. These savings were offset by greater surgeon costs and a longer hospital length of stay, which ultimately resulted in no difference in overall hospital charges [25]. Despite the shorter operative time and length of stay, the dual attending surgeries had greater screw density, which likely offsets some of the savings from the hospital admission. Therefore, a full cost analysis must be conducted prior to interpreting the financial impact of dual attending surgery on PSF for IS; many of these factors may be institution or insurance plan-specific. We do not suspect this is universal, but the way our institution handles cases designated as having "co-surgeons" is billing with modifier 62, awarding each surgeon a total of 60% of the total RVUs generated from the case.

Apart from a comparison of single-attending versus dual-attending, we additionally analyzed the value of continued surgical experience in operative outcomes for patients undergoing PSF for IS with a dual-attending approach. We found significant improvements in hospital length of stay and Hemovac blood loss with no significant differences in operative time, intraoperative blood loss, extent of instrumentation, or curve correction. However, our investigation included a total of 24 cases compiled over a four-year period. A greater investigative period may be necessary to appreciate improvements in other perioperative measures.

With a traditional single-attending approach, Cahill et al. found greater surgical experience to be associated with significant reductions in operative time, estimated blood loss, and number of levels fused [13]. Satisfaction scores measured via the Scoliosis Research Society-22 questionnaire improved with increased experience, and no difference was found in complication rates. In another investigation evaluating the role of surgeon experience during dual attending spinal surgery for idiopathic scoliosis, Chan et al. identified a learning curve of 115 cases necessary to achieve optimal operative times and 196 cases required to minimize blood loss with gradual improvements as surgeons gained experience [12]. In a separate study of patients with severe scoliosis (Cobb angle >90 degrees), 57 cases were required to achieve targeted outcomes of operative time of less than 193 minutes and total blood loss of 1612 mL [26].

Significant work has also been done on the effect of dual surgeons in the adult scoliosis deformity (ASD) population. Cheng et al. noticed a trend towards lower estimated blood loss (EBL) and similar rates of the length of stay; however, a review of the literature noted five of eight (62.5%) articles reported lower EBL, and six of eight (75%) articles reported significantly lower operation duration in dual-attending cases [27]. Our study showed similar but significant results to the current ASD literature, and while these populations can be quite different, the surgical techniques are often comparable. Traditionally, more screw density and use of interbody fusion are utilized for ASD when compared to IS surgery. As one of the junior surgeons maintains an adult deformity practice as well but utilizes a single surgeon approach, this comparison, and more importantly, the knowledge gained from it, becomes less academic. Future work of this group will include comparing the data of this surgeon's practice.

A differentiating feature of the present study is that we specifically investigated early career surgeons versus an established senior surgeon, as opposed to comparing surgeons with relatively similar experience. There is limited literature on this topic, although a recent study examining the effect on surgeon number, experience, and volume in IS patients undergoing PSF found surgical volume to be more important in perioperative outcomes rather than surgeon experience or number of surgeons [28]. They also noted that junior surgeons benefit from operating with an experienced surgeon. This highlights the importance of mentorship and teaching in surgery. As Atul Gawande has described, in order to improve at a craft, coaching is of critical importance [29]. Although two junior surgeons are not a traditional coaching approach, each individual surgeon draws on their unique training, mentors, and experiences, which can collectively help each other to keep on improving.

While the findings of our investigation demonstrate the benefits of a dual-attending approach to PSF for IS, additional factors must be taken into consideration. As a large tertiary care center with multiple fellowship-trained pediatric spine surgeons, our institution has the resources readily available to conduct dual-attending spinal deformity surgery. However, multiple attending involvement requires availability and coordination amongst attendings and services, which may not be feasible in all hospital settings. Furthermore, within an academic hospital setting, there remains a duty to train young residents. A dual-attending approach may challenge opportunities for an equivalent level of resident involvement as in a single-attending case. Regardless, the findings of our investigation show promising results of dual attending surgery for pediatric spinal deformity, including shorter operative times, better hemostasis, greater curve correction, and shorter hospital stays.

This study is not without limitations. The two cohorts were drawn from slightly different time frames. Therefore, differences in hospital policy or structure may have influenced results. Additionally, as the senior surgeon cohort utilized patients from an earlier time frame, the national trend toward earlier discharge times may have biased our findings regarding postoperative hospital stays. However, a propensity match was conducted to minimize differences between the two patient populations. This study also focused primarily on perioperative results and outcomes. Longer-term results are necessary to clarify the ultimate impact of a dual-attending approach on patient outcomes. Finally, a full cost analysis could not be conducted due to limitations in available data. Further research is needed to understand the financial implications of two attending surgeons.

## Conclusions

The results of our study suggest a dual attending approach may result in shorter operative time, greater curve correction, reduced operative blood loss, and shortened hospital length of stay. No differences were identified in postoperative blood loss or transfusion requirement, need for intensive care, or overall complication rate. Additionally, we explored the relationship between dual surgeon experience and outcomes with significant improvements in hospital length of stay and reduction in postoperative blood loss with increased experience. An understanding of the financial impact and long-term patient outcomes remains unclear and necessitates further investigation. Within the limitations of this study, we conclude that dual attending surgery in IS is safe and effective when conducted by two young orthopedic surgeons, with results that are similar to that of a more experienced senior surgeon.
